# Unraveling the dynamic changes in the intestinal microbiome: impacts on pre-weaning calf health and productivity

**DOI:** 10.1186/s40104-026-01354-6

**Published:** 2026-03-04

**Authors:** Yang Song, Shubo Wen, Le Luo Guan

**Affiliations:** 1 College of Animal Science and Technology, Inner Mongolia Minzu University, Tongliao, 028000 People’s Republic of China; 2 Department of Agricultural, Food and Nutritional Science, University of Alberta, Edmonton, T6G 2P5 Canada; 3 Department of Land and Food Systems, The University of British Columbia, Vancouver, BC V6T 1Z4 Canada

**Keywords:** Fecal microbiota transplantation, Intestinal health, Microbial colonization, Nutritional management, Pre-weaning calves

## Abstract

The early life gut microbial colonization in pre-weaning calves plays a pivotal role in shaping their health, growth, and productivity. This review delves into the dynamic changes of intestinal microbiota during early life, emphasizing key factors such as colostrum management, feeding strategies, roughage supplementation, and microbial interventions including probiotics, prebiotics, and fecal microbiota transplantation (FMT), and non-nutritional stressors that can shape the early life microbial colonization. We highlight the microbiota's critical functions in nutrient metabolism, immune development, gut barrier integrity, and gut-brain axis regulation. Additionally, the consequences of microbial dysbiosis on calf health and its long-term effects on production performance in beef and dairy cattle are discussed. While current research has provided valuable insights, understanding causal mechanisms remains a challenge. This review aims to guide practical strategies for targeted microbial management, offering a pathway to optimize early-life interventions for improved calf health and productivity.

## Introduction

The gastrointestinal tract harbors a complex and dynamic community of microorganisms, that exerts profound influences on host physiology across the lifespan. The establishment and maturation of this ecosystem during early life represents a critical developmental window with long-lasting implications for the host immune system, metabolic capacity, behavior, and nervous system development [1–3]. Understanding the mechanisms governing this foundational process is essential not only for fundamental biology but also for the practical application of improving animal health and productivity.

In pre-weaning calves, the significance of early intestinal microbial colonization is particularly pronounced. This period coincides with the development of the rumen, a specialized fermentation chamber whose functionality is dependent on its resident microbial consortium [4]. The establishment of a balanced intestinal microbiome during the pre-weaning phase lays the groundwork for efficient nutrient utilization, robust immune defenses, and optimal growth trajectories, ultimately influencing long-term productivity in both beef and dairy systems [5, 6]. Thus, the interplay among nutritional inputs, environmental factors, and microbial succession during this early window offers opportunities for targeted interventions aimed at optimizing calf health and performance.

However, unraveling the causal relationships among specific microbial shifts, host physiological development, and downstream outcomes in calves remains challenging. Significant progress has been made in characterizing microbial community structures and identifying key influencing factors such as colostrum management, starter feeding, roughage introduction, probiotics, prebiotics, antibiotics and fecal microbiota transplantation (FMT) [7–12]. These factors influence not only microbial composition but also host key physiological processes, including gut barrier maturation, immune priming, and nutrient metabolism [13–16]. Comprehensive understanding of the underlying mechanisms linking microbial dynamics to host health and productivity is still evolving. Much of the current evidence remains correlative, and translating these observations into effective management strategies requires deeper mechanistic insights.

This review synthesizes the current body of knowledge concerning the intestinal microbiome development of pre-weaning calves. We examine the process of microbial colonization, emphasizing the critical factors (nutritional and non-nutritional) that shape this ecosystem during early life. Furthermore, we will explore the functional impacts of the developing microbiome on key aspects of calf physiology, including nutrient metabolism, immune maturation, gut barrier integrity, and gut-brain axis signaling. The consequences of dysbiosis on calf health and its potential long-term effects on productive performance are also discussed. By integrating findings on colonization patterns, influencing factors, and functional outcomes, this review seeks to provide a foundation for developing evidence-based strategies for early life microbial management. Ultimately, we aim to identify opportunities for optimizing early-life interventions that promote calf health and productivity.

## Fetal gastrointestinal sterility: evidence and controversies

The traditional paradigm of fetal gastrointestinal sterility has been challenged by the molecular detection of bacterial signatures in prenatal compartments, including placental tissue, amniotic fluid, and meconium (Table 1). However, these findings warrant critical scrutiny regarding whether DNA signals reflect authentic colonization or technical artifacts.

**Table 1:** Sterility of the gastrointestinal tract in neonatal animals

Model	Method	Key findings	Controversies	Study
Cattle	16S rRNA gene sequencing	Microbiota, dominated by Proteobacteria and Firmicutes, were detected in bovine fetal tissues and fluids at 12 weeks gestation	The findings challenge the sterile womb hypothesis but face scrutiny regarding contamination risks and the biological implications of detected low-biomass microbiomes	Amat et al. [17]
Lamb	16S rRNA gene sequencing	The study found no evidence of bacterial DNA in the fetal environment or intestine during the third trimester of sheep pregnancy, reinforcing the concept of a sterile fetal environment	The findings challenge recent claims of in utero microbial colonization, sparking debates over the reliability of low-biomass DNA sequencing and the influence of contamination on reported microbiomes	Malmuthuge and Griebel [18]
Lamb	Multi-omics analysis	Detected *Escherichia coli* as predominant species in fetal lamb intestines, suggesting maternal microbial transfer	Data might be questioned due to contamination or errors in low-biomass sample analysis	Bi et al. [19]
Calves	16S rRNA gene sequencing	Fetal calf GIT and amniotic fluid harbor distinct, viable microbial communities, with colonization starting as early as 5 months of gestation	The study's findings contradict the sterile womb hypothesis and face skepticism regarding contamination control	Guzman et al. [20]
Sheep	16S rRNA gene sequencing	With strict contamination controls, this study identified trace bacterial DNA in fetal tissues, supporting the potential for maternal–fetal transfer of bacteria or their genetic material	The detection of low-biomass bacterial DNA raises concerns about contamination, and the functional significance of this DNA in fetal development remains uncertain	Rodriguez et al. [21]

### Evidence for prenatal microbial colonization​

Studies utilizing advanced molecular techniques have reported detectable microbial DNA signatures within fetal-derived specimens such as placental tissue, amniotic fluid, and meconium. Pioneer microbiota, including archaea and bacteria, have been reported to colonize in the intestine of bovine fetuses as early as d 83 of gestation, 39 archaeal and bacterial phyla were detected in the fetal calf intestine in studies employing rigorous contamination controls, including no-template controls and environmental sampling [17]. During the mid-gestation, Actinomycetales and Methanobacteriales were the most predominant bacterial and archaeal orders, respectively. In the cecal fluid and tissue of Angus × Friesian crossbred calf fetuses, suggesting distinct bacterial and archaeal communities may exist in the fetal calf gut [20]. Similarly, Husso et al. [22] detected bacterial DNA in cesarean-delivered calf meconium using deep sequencing with culture validation. Parallel observations have been reported in ovine models. *Escherichia coli* was the most predominant bacterial species detected in the fetal gut of full-term lambs delivered by aseptic hysterectomy, as determined by multi-omics analysis [19]. Maternal–fetal microbial transmission may contribute to bacterial detection in fetal lambs, as suggested by Rodriguez et al. [21], whose optimized sequencing protocols indicated in utero bacterial transfer. Collectively, these reports challenge the traditional sterile womb paradigm, suggesting potential in utero microbial transfer via placental or umbilical routes. However, these findings require critical evaluation against the contamination risks inherent in low-biomass microbiome studies.

### Methodological limitations and contamination concerns

Ongoing methodological challenges plague low-biomass research, casting significant doubt on these findings.​ Bihl et al. [23] questioned the reported presence of microbes in the fetal lamb gut by reanalyzing the original metagenomic and metatranscriptomic sequence data. They proposed that the dominant bacteria and viruses detected in the previous publication could be attributed to contamination, arising from the environment, DNA extraction kits, or experimental procedures, issues common to low-biomass microbiome studies. Malmuthuge and Griebel [18] reported that the maternal placenta, amniotic fluid, and fetal lamb intestine were sterile in late pregnancy, based on PCR, quantitative real-time PCR, and 16S rRNA gene sequencing. Kennedy et al. [24] demonstrated that most reported "fetal microbiota" signals align with common contaminants rather than biological signals. Crucially, the successful derivation of germ-free mammals via hysterectomy provides biological counter-evidence: ubiquitous fetal colonization would render such models impossible. Reanalysis of prominent studies reveals that batch effects and contamination largely explain the purported fetal-specific microbiomes.

### Current consensus

Evidence from multiple disciplines now supports the sterility of the fetal gastrointestinal tract in healthy pregnancies [24]. Methodological artifacts dominate low-biomass studies, with Kennedy et al. [25] demonstrated that > 90% of sequencing signals in cesarean-derived fetal meconium matched environmental and kit contaminants, showing compositional identity with negative controls. Walter and Hornef [26] noted that, although some studies claim the existence of a fetal intestinal microbiome, these reports often fail to adequately address methodological limitations and contamination, thereby strengthening the consensus that the fetal gastrointestinal tract is sterile in healthy pregnancies. Notably, contaminants from laboratory reagents dominate initial samples, masking biological signals that only emerge postnatally. Re-evaluation of fetal microbiome studies has attributed previously reported signals primarily to technical artifacts, including batch processing inconsistencies [27], sampling methods, sequencing technologies, and interpretation biases may have further contributed to spurious findings. Current research priorities emphasize species-specific verification (e.g., FISH/qPCR), metabolic activity assays (e.g., RNA-seq), and standardized contamination tracking across sequencing batches [24].

## Microbial colonization during the pre-weaning period

### Importance of early microbial colonization

Early microbial colonization is a dynamic process, that contributes to various physiological functions, including nutrient absorption, metabolism, intestinal immune maturation, and gut barrier development [13–15]. This process also exerts significant effects on overall health [28] and long-term productivity [29]. The early postnatal period as a critical window for implementing effective management strategies. Improved characterization of colonization patterns in neonatal ruminants, coupled with the application of targeted interventions during this period, may confer long-term health benefits.

### Colonization patterns in pre-weaning calves

#### Initial colonization after birth

**Fig. 1:**
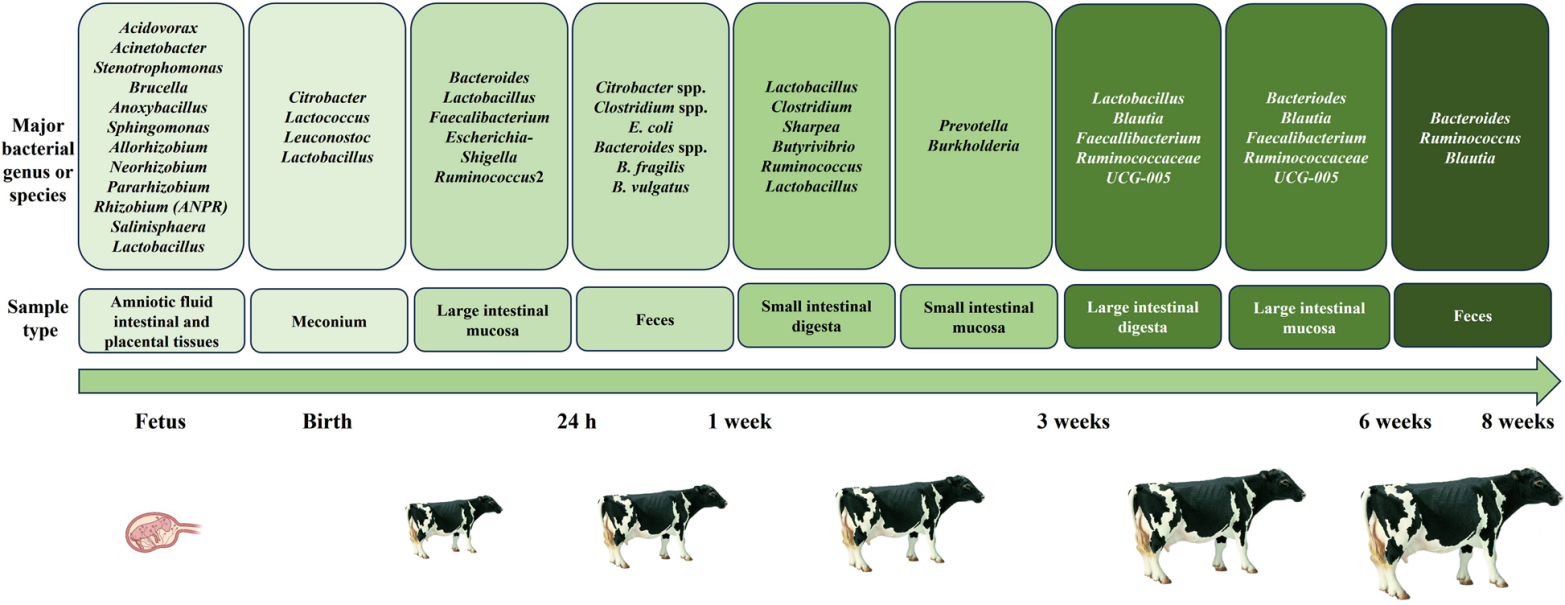
Core microbiota at genus and species level of calves. Adapted from previous reports [4, 5, 17, 30, 32]

Previous studies have suggested early microbial presence in the gut. Mayer et al. [30] identified *Leuconostoc* species as the predominant colonizers in the meconium of neonatal calves using PCR single strand conformation polymorphism (PCR-SSCP) analysis (Fig. 1). Song et al. [5] reported that mucosa-attached *Bacteroides* and *Escherichia-Shigella* were the predominant bacterial genera in the large intestine of neonatal calves. Klein-Jöbstl et al. [31] observed dynamic changes in calf fecal microbiota during the first 48 h of life, suggesting that the fecal microbiota of newborn calves may originate from the maternal birth canal. These findings indicate that bacterial communities are detectable in the calf intestine within hours of birth, with initial colonizers likely derived from maternal and environmental sources.

The intestinal microbiota of calves during the pre-weaning period exhibits dynamic changes comparable to those documented in piglets. Because the rumen is not yet functionally developed, pre-weaning calves are physiologically similar to monogastric animals. The early microbial colonization in newborn calves shows significant variation across studies [33]. Some investigations suggest that early microbial diversity is largely shaped by maternal and environmental factors, while others emphasize the role of specific feeding strategies such as colostrum or starter feed introduction. For instance, Guo et al. [34] and Dunière et al. [35] highlighted maternal influence as a primary determinant, whereas Mayer et al. [30] and Song et al. [5] reported that feeding practices exerted a more pronounced effect. These discrepancies underscore the multifactorial nature of microbial succession and highlight the need for standardized methodologies and targeted interventions.

Similar to monogastric animals, pre-weaning calves were firstly colonized by facultative anaerobic bacteria, followed by obligate anaerobes [30]. Immediately after birth, *Citrobacter*, *Lactococcus*, *Leuconostoc* and *Lactobacillus* are the initial gut colonizers in the calf meconium, potentially originating from the maternal vagina, feces and the surrounding environment. *Citrobacter* species are detected in all calves immediately after birth but disappear within 24 h. From 24 to 48 h of life, *Clostridium* species dominate the gut microbiota. Between d 1 and 7, *E. coli* becomes the predominant genus, with its abundance declining progressively between d 3 and d 7. During this period, the populations of *Bacteroides fragilis* and *Bacteroides vulgatus* increases [30].

Guo et al. [34] observed similar microbial dynamics in goat kids, where early colonizers, such as *Escherichia-Shigella* and *Citrobacter*, were primarily derived from the maternal vagina and colostrum. As the kids aged, microbial diversity increases, with *Lactobacillus* and *Butyricicoccus* becoming dominant during the non-rumination stage (7–21 d). By the transition stage (28–56 d), when solid feed intake increases, *Prevotella* and fiber-degrading bacteria (*Lachnospiraceae*, *Ruminococcaceae*) become more prevalent. In ovine models, Dunière et al. [35] reported that lambs raised by their dams exhibited a more diverse microbiota compared to artificially-reared lambs, with a delayed establishment of key fibrolytic bacteria, such as *Fibrobacter succinogenes*, particularly during the first 28 days of life. This delay in microbial colonization is influenced by the rearing system and may have significant implications for subsequent health and growth performance.

#### Changes following solid feed introduction

The introduction of calf starter initiates substantial changes in the gastrointestinal microbiota. Longitudinal studies of pre-weaning dairy calves have demonstrated that Firmicutes, followed by Bacteroidetes and Proteobacteria, dominate the fecal microbiota. These studies also report a progressive increase in bacterial richness and diversity from the first to the seventh week of life, coinciding with solid feed introduction [31, 36].

Following calf starter introduction, microbial colonization continues to evolve in both the ileum and cecum. While Firmicutes remains the predominant phylum in both regions, a shift occurs by the third week, with Firmicutes, Bacteroidetes, and Proteobacteria becoming the dominant phyla in the mucosa. At the genus level, *Prevotella* and *Burkholderia* are the most abundant genera in the mucosa of the ileum and jejunum respectively [4]. These findings indicate that microbial composition varies depending on sample type, reflecting the distinct functional niches occupied by luminal versus mucosal communities. For example, *Bacteroides* and *Prevotella* genera are involved in carbohydrate fermentation and fiber degradation [4, 8, 32], whereas *Lactobacillus* and *Bifidobacterium* contribute to immune modulation through the production of short-chain fatty acids (SCFAs) which enhance intestinal barrier integrity and attenuate inflammation [13, 37]. Recognition of these compositional and functional distinctions is fundamental to designing targeted nutritional interventions during early development.

The large intestine serves as a primary site for the fermentation of indigestible dietary substrates. Consistent with this function, Song et al. [5] reported that in 3- to 6-week-old calves, mucosa-associated taxa, including *Bacteroides*, *Blautia*, *Faecalibacterium*, and *Ruminococcaceae* UCG-005 were enriched, whereas *Lactobacillus*,* Blautia*, *Faecalibacterium*, and *Ruminococcaceae* UCG-005 predominated in the large intestinal digesta. This spatial differentiation highlights the adaptation of microbial communities to local microenvironments and functional demands.

## Factors influencing microbial colonization

**Fig. 2:**
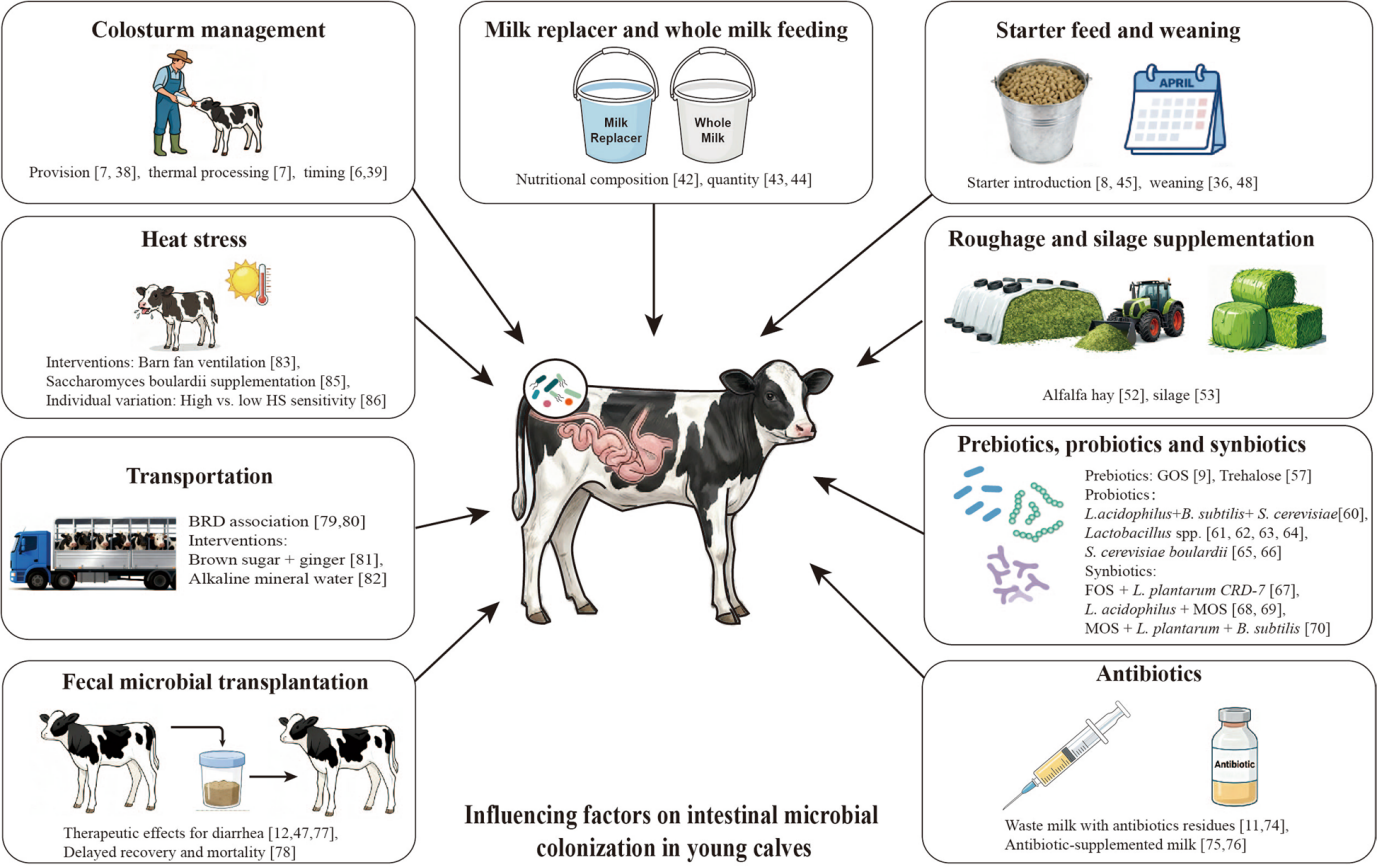
Conceptual framework of key factors shaping early-life gut microbial colonization in calves

Nutritional interventions represent a primary strategy for modulating intestinal microbial colonization in pre-weaning calves.​​ These include colostrum management, starter feed formulation, roughage/silage provision, and supplementation with prebiotics and probiotics. Collectively, these dietary inputs exert substantial influence on the establishment of the neonatal gut microbiota. Beyond nutritional factors,​​ antibiotic administration, fecal microbiota transplantation (FMT), and non-nutritional stressors, such as transportation, and heat stress also shape gut microbial community structure. Together, these interacting nutritional and non-nutritional factors form a conceptual framework for understanding early-life microbial colonization in calves, as illustrated in Fig. 2.

### Colostrum management

Colostrum is the first external nutrient source for neonatal calves. Beyond its recognized role in passive immunity transfer, colostrum feeding practices shape the early intestinal microbiota through the provision of colostrum, colostrum feeding time and its thermal processing. The act of colostrum feeding, independent of processing method, appears to favor the establishment of specific bacterial populations. Song et al. [38] reported that calves receiving fresh colostrum harbored higher proportions of *Clostridium* cluster XIVa and *Bifidobacterium*, alongside reduced *E. coli* abundance, compared to colostrum-deprived controls. *Clostridium cluster* XIVa contributes to butyrate production, while *Bifidobacterium* is associated with competitive exclusion of pathogens. A parallel analysis from the same trial by Malmuthuge et al. [7] demonstrated that heat-treated colostrum also elevated *Bifidobacterium* and suppressed *E. coli* in the small intestine. The likely explanation is that pasteurization reduces viable pathogen load while preserving milk oligosaccharides and immunoglobulins that selectively support commensal bacteria. These observations, however, derive from a single experimental cohort with modest sample sizes, and whether such compositional shifts persist beyond the neonatal period or translate into improved health outcomes needs confirmation in larger, longitudinal studies.

The timing of first colostrum feeding constitutes an additional determinant of early microbial colonization. Fischer et al. [6] demonstrated that delaying colostrum administration to 12 h post-birth attenuated mucosal attachment of *Bifidobacterium* spp., *Lactobacillus* spp., and *E. coli* in calves examined at 2 days of age, relative to counterparts fed within the first hour. Extended analyses from the same cohort revealed that delayed feeding perturbed mucosa-associated communities throughout the ileum and colon [39], coinciding with differential expression of host genes implicated in innate immune signaling and epithelial barrier maintenance [40, 41]. The neonatal mucosa likely undergoes a transient period of heightened receptivity to bacterial adhesion, reflecting immature barrier function and available epithelial binding sites. Delayed colostrum feeding may truncate this window, limiting pioneer colonizer establishment.

### Milk replacer and whole milk feeding

Following the colostrum period, liquid feed—whether milk replacer or whole milk—becomes the primary nutrient source for pre-weaning calves. Emerging evidence suggests that both the nutritional composition and the quantity of liquid feed may influence intestinal microbial colonization [42, 43]. With respect to composition, Badman et al. [42] reported that milk replacer containing 74% crude protein from whey protein phospholipid concentrate, enriched with conjugated milk oligosaccharides, promoted *Bifidobacterium* and *Faecalibacterium prausnitzii* colonization by d 7. And the beneficial effect is attributed to oligosaccharide-mediated pathogen suppression and enhanced short-chain fatty acid production. Additionally, the amount of milk replacer or whole milk provided, also affects the intestinal microbiota and its metabolites [43, 44]. Greater bacterial diversity and three-fold higher *Faecalibacterium* abundance in calves offered ad libitum milk replacer compared to those restricted to 10% body weight were observed [43]. Similarly, Alimirzaei et al. [44] found elevated *Lactobacillus* and reduced *Escherichia coli* in calves receiving whole milk at 20% compared to 10% body weight during the first three weeks. Higher nutrient availability likely favors fermentative bacteria while suppressing opportunistic pathogens. However, confounding factors under ad libitum regimens, such as altered gut transit and luminal pH, complicate interpretation, and the relative contributions of oligosaccharides versus protein source remain to be determined.

### Starter feed and weaning

The introduction of solid feed marks a pivotal nutritional shift during the pre-weaning period. Starter feed, typically a grain-based formulation providing starch, protein, and fiber, is generally offered from approximately 7 days of age to stimulate rumen development. This dietary transition reshapes the intestinal microbial landscape. In yak calves, starter supplementation enriched starch-degrading taxa such as *Prevotella*, *Synergistes*, and *Sphingomonas*, coinciding with elevated cecal volatile fatty acid concentrations and improved growth performance [8]. In dairy calves, starter introduction increased bacterial phylotype diversity and upregulated mucosal immune gene expression, though total bacterial density remained unchanged [45]. These findings indicate coordinated microbiota-host responses to dietary transition. However, direct causal relationships between specific taxa and functional outcomes were not established in either study.

Beyond starter introduction, the weaning transition further reshapes gut microbial assembly. As calves shift from milk-starter combinations to starter and forage, the gut microbiota undergoes reorganization favoring fibrolytic and saccharolytic taxa. In the feces of 54-day-old weaning calves, Bacteroidetes (44.8%) and Firmicutes (41.8%) were identified as dominant phyla, with *Bacteroides* (17.78%), *Ruminococcus* (10.51%), and *Blautia* (9.82%) prevailing at genus level [32]. However, the persistence of such diet-induced changes remains uncertain, as early dietary interventions altered rumen microbiota but these shifts did not persist into adulthood [46]. Notwithstanding this limitation, these genera play recognized roles in complex carbohydrate degradation [5], a function consistent with the metabolic demands of the weaning transition [47]. Moreover, a study by Scully et al. [48] suggested the *Bacteroidetes*–*Firmicutes* dominance represents a conserved post-weaning feature. Furthermore, confounding between dietary composition and nutrient intake, coupled with absent clinical endpoints, leaves the functional significance of these shifts unresolved.

### Roughage and silage supplementation

Given the limited fermentative capacity of the developing rumen, roughage consumed during the pre-weaning period is largely digested by hindgut microbiota [5, 49], positioning the large intestine as the primary site for fiber fermentation in young calves. Available evidence suggests that hay supplementation enriches fiber-degrading taxa. In yak calves, alfalfa hay increased cellulolytic and hemicellulolytic genera such as *Eubacterium*, *Psychrobacter*, *Butyrivibrio*, and *Lachnospira*, with concurrent elevations in cecal acetate and propionate [8]*.* The proposed mechanism centers on volatile fatty acid production, which has been implicated in epithelial barrier maintenance [50], and colonocyte proliferation [51]. Consistent with this, early oral fiber administration promoted *Lactobacillus* while suppressing *Clostridium perfringens*, and was associated with greater body weight at 21 d [52]*.* Similar modulatory effects have been observed with silage supplementation. Silage supplementation may exert similar effects. Calves receiving silage achieved microbiota compositions more closely resembling adult cattle by weaning [53]. suggesting accelerated community maturation. However, direct causal evidence linking these microbial shifts to health outcomes remains limited, and whether adult-like composition confers functional advantages has not been established. Moreover, silage quality depends on feedstock and fermentation conditions, and this variability warrants standardization in future studies.

### Prebiotics, probiotics, and synbiotics

Apart from the dietary factors discussed above, supplementation with prebiotics, probiotics, and synbiotics has been investigated as a means to modulate gut microbial colonization in pre-weaning calves.

#### Prebiotics

Prebiotics promote short-chain fatty acid and bacteriocin production, contributing to pathogen suppression [54]. These non-digestible substrates resist host enzymatic degradation but are selectively fermented by beneficial microbes [55]. Galactooligosaccharide (GOS)-enriched milk replacers supplementation increased *Lactobacillus* and *Bifidobacterium* abundance in Holstein calves [9], consistent with the selective fermentation hypothesis [56]. Trehalose supplementation reduced potentially pathogenic *Clostridium* species while elevating butyrate concentrations in 3-week-old calves [57], with butyrate implicated in intestinal barrier integrity and immune modulation [58]. Nevertheless, prebiotic efficacy depends on dosage and substrate selection, and optimal supplementation regimens have not been established. Whether these effects persist beyond the supplementation period remains unexplored.

#### Probiotics

Probiotics, including bacterial and yeast strains, are widely used in calf production, with approximately 41% of pre-weaning calves receiving supplementation [59]. Evidence suggests strain-specific effects on gut microbiota composition and health outcomes. Multispecies formulations containing *Lactobacillus acidophilus*, *Bacillus subtilis*, and *Saccharomyces cerevisiae* increased beneficial taxa while reducing *Bacteroidetes* and diarrhea incidence [60]. Single-strain *Lactobacillus* supplements have shown similar patterns. *Lactobacillus johnsonii* TP1.6 or *Limosilactobacillus reuteri* TP1.3B elevated *Bifidobacterium* and *Akkermansia* in calf feces [61]. While *Limosilactobacillus reuteri* BF-E7 and *Ligilactobacillus salivarius* BF-17 enhanced *Lactobacillus* and *Bifidobacterium* populations and fecal lactate concentrations in buffalo calves [62]. Heat-killed *Lactobacillus sakei* HS-1 reduced *Escherichia coli* loads and medical interventions in Japanese Black calves [63], and oral *Lactobacillus reuteri* administration conferred protection against neonatal diarrhea [64].

Yeast-based probiotics have also demonstrated modulatory potential. *Saccharomyces cerevisiae boulardii* (SCB) supplementation at 5 g/d increased ileal species richness and phylogenetic diversity in Holstein calves, correlating with elevated secretory immunoglobulin A (sIgA) secretion [65]. SCB also stimulated fecal lactobacilli populations and altered colon morphology, including reduced crypt depth and enhanced mucin production [66]. Despite these promising observations, variability in probiotic strains, dosages, and host breeds across studies complicates direct comparisons and limits the generalizability of current findings.

#### Synbiotics

Synbiotics combine prebiotics and probiotics to theoretically enhance efficacy through complementary mechanisms. Fructooligosaccharide combined with *Lactobacillus plantarum* CRD-7 increased fecal *Lactobacillus* and *Bifidobacterium* while reducing diarrhea incidence by 42% [67]. Mannan-oligosaccharides (MOS) with *Lactobacillus acidophilus* decreased fecal *E. coli* counts and improved body weight gain in crossbred calves [68], with similar microbial shifts observed in Murrah buffalo calves over 120 d [69]. MOS combined with *Bacillus subtilis* yielded 78 g greater daily weight gain between 42 and 56 days of age [70]. These observations indicate that synbiotic supplementation may benefit both gut health and growth performance in pre-weaning calves. Nevertheless, the additive or synergistic nature of these effects has rarely been tested against single-component controls.

### Antibiotic exposure

Pre-weaning calves may be exposed to antibiotics through multiple routes, including therapeutic treatment for diarrhea or respiratory disease, and consumption of waste milk from cows undergoing antibiotic therapy. While waste milk offers economic advantages, its effects on intestinal microbiota warrant careful consideration. Studies examining rumen microbiota reported only minimal changes in calves fed waste milk at 49 and 63 days of age [71], but the impact on hindgut communities has received less attention. Waste milk may contain both pathogenic microorganisms and residual antibiotics. Although pasteurization eliminates most pathogens, antibiotic residues can persist and potentially disrupt beneficial microbial populations [72]. Calves receiving waste milk from cows treated for intramammary infections exhibited reduced abundance of *Faecalibacterium* alongside increased *Campylobacter*, *Pseudomonas*, and *Chlamydophila*, with higher diarrhea incidence [73]. Even low antibiotic concentrations can inhibit beneficial taxa such as *Bifidobacterium* and *Faecalibacterium prausnitzii* [74], suggesting that subtherapeutic exposure may carry underappreciated consequences.

Experimental studies have further characterized antibiotic-induced microbial perturbations. Milk supplemented with antibiotics produced significant compositional differences at the genus level in fecal microbiota [11]. Low concentrations of antibiotic mixtures containing penicillin, streptomycin, tetracycline, and ceftiofur in milk replacers induced similar genus-level alterations [75]. In Japanese Black calves, chlortetracycline supplementation reduced the relative abundance of butyrate-producing *Lachnospiraceae* while increasing methanogenic taxa at 60 days of age [76], raising potential concerns regarding both gut health and environmental impact. Antibiotic exposure disrupts gut microbial development in pre-weaning calves, but the durability and health implications of these changes remain unclear due to short study durations and confounding nutritional factors.

### Fecal microbiota transplantation

Fecal microbiota transplantation (FMT) has been investigated as a therapeutic approach for diarrhea in pre-weaning calves, based on the premise that transferring gut microbiota from healthy donors can restore microbial homeostasis in diseased recipients. A trial with 57 calves suggested increased *Porphyromonadaceae* abundance and decreased amino acid concentrations following FMT, with shifts in microbial composition and metabolite profiles correlating with clinical improvement [12]. Another study observed elevated proportions of *Blautia*, *Lactobacillus*, *Ruminococcus*, and *Romboutsia* in Chinese Holstein calves, alongside reduced diarrhea incidence at a dose of 1 × 10^8^ CFU/mL [47]. Pre-treatment abundance of *Sporobacter* and reduced concentrations of glycerol-3-phosphate and dihydroxyacetone phosphate have been proposed as predictive markers of treatment success [77].

However, conflicting outcomes limit confidence in FMT's therapeutic value. In one cohort, recipients displayed microbial community alterations linked to delayed recovery and elevated mortality [78]. Explanations for these disparities likely include heterogeneity in donor selection criteria, fecal processing methods, recipient baseline health, and underlying diarrhea etiology. Inconsistent protocols for material preparation, storage, and dosing across studies complicate efforts to replicate findings [12, 77]. Most trials have used small cohorts with limited follow-up, precluding assessment of long-term outcomes or rare adverse events. Given the variable natural history of calf diarrhea, large-scale controlled studies are needed to establish FMT's efficacy before clinical use.

### Environmental stressors

Beyond dietary factors, environmental stressors such as transportation and heat exposure can disrupt gut microbial communities in pre-weaning calves. Transportation stress has been associated with bovine respiratory disease (BRD), with bovine coronavirus (70.1%) and *Histophilus somni* (86.6%) identified as prevalent pathogens in transported cattle [79]. The duration of transportation also modulates microbial communities, calves transported for 16 h exhibited significantly greater reductions in Fusobacteria abundance compared to those transported for 6 h, accompanied by decreased *Lactobacillus* and increased *Bacteroides* and *Butyricicoccus* over time [80]. Post-transport interventions may mitigate these effects. Placement strategies incorporating brown sugar and ginger supplementation reduced BRD-associated pathogen abundance [81], while alkaline mineral water administration improved health indicators and decreased *H. somni* colonization prior to transport [82]. These findings indicate that strategic interventions before and after transportation can effectively mitigate transport-induced microbial dysbiosis and reduce BRD susceptibility in calves.

Heat stress (HS) also affects respiratory and gastrointestinal microbial communities in neonatal calves. Ventilation via barn fans reduced the mean relative abundance of *Mycoplasma* and *Moraxella* at 5 and 9 weeks, respectively [83], genera previously linked to pneumonia in calves [84]. HS has been associated with increased diarrhea, elevated rectal temperature and heart rate, and higher abundance of *Escherichia coli* and *Enterobacteriaceae* in the gut. Supplementation with *Saccharomyces boulardii* reduced these effects [85], though whether this reflects direct microbial modulation or indirect metabolic buffering requires further investigation. Individual variation in HS responses has also been documented. Calves with high versus low HS sensitivity exhibited differences in gastrointestinal microbiota and serum metabolite profiles, particularly in the abundance of *Ruminococcus* and *Olsenella* and concentrations of malic acid and fumaric acid [86]. These biomarkers may serve as predictive indicators, although their mechanistic relationship to HS tolerance is not yet established.

## Functional roles of early-life microbiota

The intestinal microbiota established during the pre-weaning period has been implicated in multiple aspects of calf physiology, including nutrient digestion, immune development, intestinal barrier function, energy metabolism, and gut-brain axis maturation [28, 87]. Disruptions to microbial communities during this period have been associated with health complications and reduced productivity, though mechanistic understanding in ruminants remains limited. Much functional evidence derives from germ-free mouse models, but ruminants' distinct gastrointestinal anatomy and developmental trajectories complicate cross-species extrapolation. Direct investigations in calves are scarce, and whether rodent-derived mechanisms apply to ruminants remains untested. Below, we review proposed functional roles of early-life microbiota in calves, acknowledging the predominance of correlative evidence.

Taken together, evidence from nutritional interventions, microbial manipulations, and environmental stressors demonstrates that early-life gut microbial colonization in calves is shaped by a wide range of interacting factors. Representative studies supporting these influences, including experimental conditions and reported microbial responses, are summarized in Table 2.

**Table 2:** The main factors affect the microbial colonization of young calves

Factors	Age and breed	Treatment	Conclusions
Colostrum	Holstein calves at 6 h and 12 h after birth	Calves were in three groups: fresh colostrum (FC, *n* = 12); heat-treated colostrum (HC, 60 °C for 60 min, *n* = 12); no colostrum (NC, *n* = 8)	Fresh and heat-treated colostrum feeding increased *Bifidobacterium* and inhibited *E. coli* colonization in the colon of newborn calves [38]
	Holstein calves at 6 h and 12 h after birth	Calves were in three groups: fresh colostrum (FC, *n* = 12); heat-treated colostrum (HC, 60 °C for 60 min, *n* = 12); no colostrum (NC, *n* = 8)	Heat-treated colostrum feeding enhanced tissue-attached *Bifidobacterium* colonization while reducing *E. coli* colonization in the small intestine of calves [7]
	Holstein calves at 51 h after birth	Calves (*n* = 27) were randomly assigned to three groups: fed colostrum at 45 min (0 h, *n* = 9), 6 h (*n* = 9), or 12 h (*n* = 9) after birth	Delayed colostrum feeding by 6 or 12 h reduced IgG absorption efficiency and delayed mucosa-attached *Bifidobacterium* spp., *Lactobacillus* spp., and *E. coli* colonization [6]
	Holstein calves at 51 h after birth	Calves (*n* = 27) were randomly assigned to three groups: fed colostrum at 45 min (0 h, *n* = 9), 6 h (*n* = 9), or 12 h (*n* = 9) after birth	Delaying first colostrum feeding to 12 h after birth significantly increased the relative abundance of ileum mucosa-associated *Enterococcus* and *Streptococcus*, while reducing colon mucosa-associated *Lactobacillus* [39]
Milk replacer/whole milk	Holstein–Friesian dairy calves at 0, 7, 14, 28, and 49 d after first consumption of milk replacer	Calves (*n* = 10) were assigned to two groups fed different milk replacers: MR1 (*n* = 5, 55% crude protein from whey, higher free milk oligosaccharides) or MR2 (*n* = 5, 74% crude protein from whey protein phospholipid concentrate, higher conjugated milk oligosaccharides)	Calves fed MR2 showed significantly greater relative abundance of *Bifidobacterium* spp. and *Faecalibacterium prausnitzii* at d 7 compared to MR1-fed calves [42]
	Kiwi cross calves (Holstein–Friesian x Jersey) at pre-weaning (67 ± 3 d on trial)	Calves (*n* = 199) were assigned to three milk replacer allowance groups using automated feeders: low allowance (LA, 10% of initial body weight/d, *n* = 67), high allowance (HA, 20% of initial body weight/d, *n* = 65), or ad libitum (ADLIB, *n* = 66)	Higher milk replacer allowances increased hindgut bacterial diversity, and abundances of beneficial *Faecalibacterium spp. *[43]
	Holstein female calves from birth to 98 d of age	Calves (*n* = 48) were randomly assigned to two groups: high milk (HM, *n* = 24, 20% of birth BW for the first 3 weeks, then gradually reduced to 10% until weaning on d 51) or low milk (LM, *n* = 24, 10% of birth BW throughout). Fecal samples were collected on d 7, 14, and 21	Higher milk allowance improved fecal *Lactobacillus,* and inhibited *Escherichia coli.* Higher milk allowance calves exhibited lower serum TNF-α and cortisol levels on d 14 and 28, fewer days with fever and greater body weight at d 21, 56, and 98 [44]
Calf starter	Male yak calves (*Bos grunniens*) from 30 to 120 d of age	Calves (*n* = 20) were randomly assigned to four groups: milk replacer only (CON, *n* = 5), milk replacer + alfalfa hay (A, *n* = 5), milk replacer + starter feed (S, *n* = 5), or milk replacer + starter feed + alfalfa hay (SA, *n* = 5)	Co-supplementation with alfalfa hay and starter feed (SA group) significantly improved growth performance and increased caecal VFA concentrations while maintaining immune homeostasis. While starter feed alone (S group) significantly increased caecal lactate and LPS contents, leading to elevated plasma cortisol, NO, TNF-α, and IFN-γ levels indicative of inflammation [8]
	Holstein bull calves at 49 ± 5.2 d of age	Calves (*n* = 8) were paired by BW and assigned to two groups: milk replacer only (MR, 750 g/d, *n* = 4) or milk replacer + calf starter (MR + S, starter ad libitum, *n* = 4)	Calf starter feeding tended to increase bacterial phylotype richness along the GIT and significantly altered mucosal immune gene expression [45]
Roughage	Holstein calves from 3 d to weaning (50 d)	Calves (*n* = 20) were divided into control (*n* = 10) or treatment (*n* = 10) groups. Treatment calves received oral administration of ground timothy hay and psyllium (50:6 ratio) at 50 g/d from 3–7 d and 100 g/d from 8 d until weaning. Fecal samples were collected at 7, 21, 35, 49, and 56 d	Oral fiber administration improved ADG during the first 21 days of life and increased fecal propionate proportion at 7 d. *Lactobacillus* and *Prevotellaceae* were enriched at 7 d, while *C. perfringens* detection was reduced [52]
	Holstein dairy cows from 2 weeks to first lactation cycle (~ 2 years)	Holstein calves (*n* = 45) were assigned to three groups: calf starter grains, corn silage, or a 25:75 starter/silage mixture	Significant diet-associated differences in fecal archaeal and bacterial communities were observed by weaning (8 weeks), with silage-fed calves achieving a more adult-like microbiota composition earlier than starter-fed calves [53]
Prebiotics/Probiotics/Synbiotics	Holstein calves from 1–4 d to 8 weeks of age	Calves (*n* = 80) were randomly assigned to two groups: control milk replacer (CON, *n* = 40) or GOS-supplemented milk replacer (GOS, *n* = 40). At 2 and 4 weeks, 8 calves per treatment were euthanized for intestinal digesta and tissue collection. The remaining 48 calves continued to week 8	GOS supplementation significantly increased colonic *Lactobacillus* and *Bifidobacterium* relative abundance at 2 weeks of age, while reducing *Fecalibacterium*, *Oscillospira*, and *Clostridium*. Additionally, GOS-fed calves showed greater intestinal villus length in duodenum, jejunum, and deeper colonic crypts, but also exhibited lower colonic digesta DM [9]
	Crossbred calves (Japanese Black × Holstein) from approximately 11–20 d of age to weaning (~ 72 d)	Experiment 1: Calves (*n* = 173) were divided into control (*n* = 83) and trehalose (*n* = 90) groups. Experiment 2: Male calves (*n* = 20) were divided into control (*n* = 10) and trehalose (*n* = 10) groups	Trehalose significantly reduced medication frequency, reduced *Clostridium* spp. and enriched fiber-degrading bacteria *Prevotella* and *Lachnospira* by weaning [57]
	Chinese Holstein female calves from 6 ± 3 d to 8 weeks of age	Calves (*n* = 40) were assigned to four groups: control (C, *n* = 10), T1 (0.5 g MSP/calf/d, *n* = 10), T2 (1 g MSP/calf/d, *n* = 10), or T3 (2 g MSP/calf/d, *n* = 10). MSP contained *Lactobacillus acidophilus* (3 × 10^9^ CFU/g), *Bacillus subtilis* (3 × 10^9^ CFU/g), and *Saccharomyces cerevisiae* (1 × 10^9^ CFU/g). Fecal samples were collected at week 2, week 4, week 6 and week 8	High-dose MSP (2 g/d, T3) increased *Bifidobacterium*, *Lactobacillus*, and *Collinsella* abundances and reduced diarrhea incidence in pre-weaning calves at week 2 [60]
	Holstein calves from 5–9 d to 21 d	Calves (*n* = 30) were assigned to three groups: control (*n* = 10), *Limosilactobacillus reuteri* TP1.3B (*n* = 10), or *Lactobacillus johnsonii* TP1.6 (*n* = 10)	High relative abundance of *Bifidobacterium* and *Akkermansia* were displayed in both treated groups compared to control [61]
	Murrah buffalo calves from 5–7 d to 60 d of age	Calves (*n* = 16) were in four treatments: control (CT, *n* = 4), *Limosilactobacillus reuteri* BF-E7 (LR, 1 × 10^8^ CFU/g/d, *n* = 4), *Ligilactobacillus salivarius* BF-17 (LS, 1 × 10^8^ CFU/g/d, *n* = 4), or consortium of both strains (CS, 1 × 10^8^ CFU/g/d, *n* = 4)	Probiotics supplementation increased dry matter intake (DMI, g/d), average daily gain, net body weight gain, feed conversion efficiency, and structural growth measurements, as well as the relative abundance of *Lactobacillus* and *Bifidobacteria* compared to control [62]
	Japanese Black calves from 2–12 d to 3 weeks after separation	Calves (*n* = 10) were in two treatments: control (*n* = 5) or HK-LS HS-1 supplement (*n* = 5)	HK-LS HS-1 supplementation significantly increased fecal lactic acid bacteria counts on d 21 and reduced medication frequency and treatment costs compared to control [63]
	Newborn Simmental calves from 2 to 14 d of age	Calves (*n* = 166) from 10 dairy farms were assigned to two groups: control and *L. reuteri* group	*L. reuteri* administration significantly reduced diarrhea incidence within the first 2 weeks of life, with the protective effect most pronounced between d 3 and 10 [64]
	Male Holstein calves from birth to 1 week of age	Calves (*n* = 20) were randomly assigned to two groups: control (CON, *n* = 10) or SCB (*n* = 10, 5 g/d live *S. cerevisiae boulardii* CNCM I-1079, 10 × 10^9^ CFU/d). At 1 week, calves were euthanized and digesta/tissue samples from proximal jejunum, ileum, and colon were collected	SCB supplementation significantly increased species richness and phylogenetic diversity in ileum digesta. SCB enriched *Eubacteriaceae, Corynebacteriaceae, Eggerthellaceae, Bacillaceae,* and *Ruminococcaceae* families [65]
	Dairy calves from 2–7 d to 96 d of age	Calves (*n* = 32) were randomly assigned to four groups: control (CTL, *n* = 8), *S. cerevisiae boulardii* CNCM I-1079 (SCB, 7.5 × 10^8^ CFU/L milk replacer + 3 × 10^9^ CFU/kg starter, *n* = 8), *L. acidophilus* BT1386 (LA, 2.5 × 10^8^ CFU/L milk replacer + 1 × 10^9^ CFU/kg starter, *n* = 8), or antibiotic growth promoter (ATB, chlortetracycline + neomycin, *n* = 8). Digesta from rumen, ileum, and colon, and mucosa from ileum and colon were collected at d 33 (pre-weaning) and d 96	Both SCB and LA reduced pathogenic *Streptococcus* and *Tyzzerella*_4 and increased beneficial *Fibrobacter*, with effects predominantly in the ileum during pre-weaning. SCB specifically enriched *Roseburia* and *Olsenella*. SCB and LA had similar impact on diversity as ATB but more diverse effects on bacterial composition [66]
	Murrah buffalo calves from 5 to 80 d of age	Calves (*n* = 24) were divided into four groups: control (CON, *n* = 6), SYN1 (3 g FOS + *L. plantarum* CRD-7 in 150 mL fermented milk, *n* = 6), SYN2 (6 g FOS + *L. plantarum* CRD-7 in 100 mL fermented milk,* n* = 6), or SYN3 (9 g FOS + *L. plantarum* CRD-7 in 50 mL fermented milk, *n* = 6). Synbiotics were administered daily for 75 d	Calves in SYN3 displayed improved digestibility, antioxidant enzymes, and immune status, along with increased *Lactobacilli* and *Bifidobacterium* counts and reduced diarrhea incidence [67]
	Crossbred (Sahiwal × Holstein Friesian) calves from 15 to 105 d of age	Calves (*n* = 24) were divided into four groups: control (T0, *n* = 6), probiotic (T1,* n* = 6, *L. acidophilus* 2 × 10^10^ CFU/g @ 1 g/calf/d), prebiotic (T2, *n* = 6, MOS 4 g/calf/d), or synbiotic (T3, *n* = 6, *L. acidophilus* 0.5 g + MOS 2 g/calf/d). Additives were mixed in milk and fed for 90 d	Probiotic and synbiotic groups showed significantly higher total body weight gain and DM digestibility. All treatment groups reduced fecal coliform and *E. coli* counts at d 15 and 30 [68]
	Murrah buffalo calves from 5–7 d to 120 d of age	Calves (*n* = 20) were divided into four groups: control (CON, n = 5), prebiotic (PRE, *n* = 5, MOS 4 g/calf/d), probiotic (PRO, *n* = 5, *L. acidophilus* fermented milk 200 mL/d containing 10^8^ CFU/mL), or synbiotic (SYN, *n* = 5, MOS + *L. acidophilus* at same doses). Fecal samples were collected at d 0, 30, 60, 90, and 120	Synbiotic supplementation significantly improved ADG and NDF digestibility compared to control. All treatments increased fecal *Lactobacillus* and *Bifidobacterium* while reducing coliform counts. Fecal pH and ammonia decreased, while VFA (acetate, propionate, butyrate) increased. Synbiotic showed greatest effects, suggesting MOS and *L. acidophilus* combination optimally improves performance and gut health [69]
	Holstein heifer calves from 4–12 h of age to weaning at 60 d	Calves (*n* = 1801) were in four groups: control (CON, *n* = 450), prebiotic (PRE, 14 mL yeast culture enriched with MOS, *n* = 450), probiotic (PRO, 1 g *Bacillus subtilis* + *Lactobacillus plantarum*, 1 × 10^9^ + 2.5 × 10^8^ CFU/d, *n* = 451), or synbiotic (SYN, combination of PRE and PRO, *n* = 450)	Synbiotic supplementation increased overall ADG by 19 g/d compared to control. During late preweaning (42–56 d), PRE and SYN increased ADG by 85 and 78 g/d, respectively. Probiotic reduced *Cryptosporidium* shedding 100-fold at 14 d. Prebiotic reduced fecal *E. coli* and pathogenic *E. coli* at 42 d [70]
Antibiotics	Male Holstein calves from birth to 6 weeks of age	Calves (*n* = 30) were randomly assigned to two groups: no drug residues (NR, *n* = 15, raw milk without antimicrobials) or drug residues (DR, *n* = 15, raw milk spiked with ceftiofur 0.1 μg/mL, penicillin G 0.005 μg/mL, ampicillin 0.01 μg/mL, and oxytetracycline 0.3 μg/mL). Fecal samples were collected weekly from birth (pre-treatment) to 6 weeks	Discriminant analysis showed clear separation between DR and NR calves at the genus level from week 1 onwards, indicating that drug residues at FDA tolerance levels affect gut microbiota composition. Additionally, *Clostridium* (*P* = 0.03) and *Streptococcus* (*P* = 0.004) were significantly reduced in DR calves [11]
	Male Holstein calves from birth to 6 weeks of age	Calves (*n* = 14) were assigned to two groups: control (NR, *n* = 7, raw milk without antimicrobials) or drug residues (DR, *n* = 7, raw milk spiked with ceftiofur 0.1 μg/mL, penicillin G 0.005 μg/mL, ampicillin 0.01 μg/mL, and oxytetracycline 0.3 μg/mL). Calves were bucket-fed one gallon of milk twice daily. Fecal samples were collected at weeks 0, 1, 3, and 6	Drug residues significantly altered fecal microbiota functional profiles, with decreased "Stress Response", "Regulation and Cell Signaling", and "Nitrogen Metabolism" genes in DR calves at week 1. Drug residues also resulted in more homogeneous RATC profiles over time, suggesting selective pressure on resistance gene distribution [74]
	Holstein calves from 1 to 35 d of age	Calves (*n* = 12) were assigned to three groups: control (CON, *n* = 4, milk replacer without antibiotics), low cocktail of antibiotics (LCA, *n* = 4, penicillin 0.024 mg/L + streptomycin 0.025 mg/L + tetracycline 0.1 mg/L + ceftiofur 0.33 mg/L), or low single antibiotic (LSA, *n* = 4, ceftiofur 0.33 mg/L only). Antibiotics were added to MR and fed twice daily. At 35 d, calves were euthanized and digesta from ileum, colon, and rectum were collected	LCA significantly reduced ileal *Enterobacteriaceae* and *Escherichia coli*, while LSA reduced *Comamonas*. In the rectum, both LCA and LSA reduced *Acidaminococcaceae* and *Phascolarctobacterium* [75]
	Japanese Black calves from 3 to 60 d of age	Calves (*n* = 12) were assigned to CON (*n* = 6, milk replacer containing CTC at 10 g/kg) or EXP (*n* = 6, antibiotic-free milk replacer). Fecal samples were collected at 3, 30, and 60 d	CTC altered weighted UniFrac distances at 60 d and increased methanogens *Methanobrevibacter*, while antibiotic-free calves showed higher *Lachnospiraceae* [76]
Fecal transplantation	Korean brown cattle (*Bos taurus coreanae*) calves aged 5–50 d with moderate-to-severe diarrhea	Diarrheic calves (*n* = 57) were assigned to three groups: control (CON, *n* = 14, saline), antibiotic (ABX, *n* = 23, neomycin ± other antibiotics), or FMT (*n* = 20, 5 g feces as bolus at 0.1 g/mL, administered orally 5 times). Healthy donor calves (*n* = 6, aged 21–50 d) were rigorously screened. Fecal samples were collected at d 0, 2, 4, 8, 16, 32, and 48	FMT achieved 95% complete remission rate vs. 35.7% (CON) and 26.1% (ABX), with 0% mortality vs. 14.3% and 17.4%. FMT increased *Porphyromonadaceae* abundance, which negatively correlated with diarrhea (*r* = −0.714, *P* = 0.041), while reducing *Enterobacteriaceae* [12]
	Chinese Holstein calves from 50 d (weaning) to 80 d of age	Calves (*n* = 50) were divided into five groups: NC (*n* = 10, no supplementation), Control (*n* = 10, 5 mL saline), LFMT (*n* = 10, 5 mL of 1 × 10^8^ CFU/mL fecal suspension), HFMT (*n* = 10, 5 mL of 1 × 10^9^ CFU/mL), or SFMT (*n* = 10, sterilized fecal suspension). Treatments were administered orally every other day from d 50 to 60. Fecal samples were collected at d 5, 10, 15, and 20 post-weaning	FMT significantly enhanced the relative abundance of beneficial bacteria, such as *Blautia*, *Lactobacillus, Ruminococcus* and *Romboutsia.* Diarrhea rates were significantly reduced, with the LFMT group showing the most pronounced effect [47]
	Holstein, Jersey, and Jersey-cross heifer calves from 4–12 d to 21 d of age	Calves (*n* = 227) were assigned to control (*n* = 115, standard farm protocol) or FMT (*n* = 112, ~ 36 g processed fecal matter orally once daily for 3 d)	Calves were less likely to recover from diarrhea and more likely to die after FMT. Higher relative abundance of *Lactobacillus* and *Lactobacillus reuteri* and lower relative abundance of *Clostridium nexile* and *Bacteroides vulgatus* on d 10 were detected [77]
	Health and diarrheic calves, and the trail lasted for 7 d after FMT	Twenty FMT treatments were conducted: healthy donors (*n* = 20) and diarrheal recipients (*n* = 20) were selected from the same farm	*Sporobacter* genus and metabolites such as glycerol 3-phosphate, dihydroxyacetone phosphate, and isoamylamine could serve as biomarkers for donor selection [78]
Transportation	Limousine beef steers, approximately of 6–10 months	Nasopharyngeal swabs were collected from 231 calves at three time points: before transportation from the origin farm, within four days of arrival at the feedlot, and during clinical examination of each calf at the feedlot	Bovine coronavirus (BCoV, 70.1%) and *Histophilus somni* (86.6%) were the most prevalent viral and bacterial pathogens in nasopharyngeal swabs of transported cattle, with a significant association observed between *Mannheimia haemolytica* positivity and respiratory clinical signs. The nasopharyngeal microbiota was significantly affected by long-distance transportation, suggesting that enhancing calf immunity prior to loading may be more effective in reducing BRD risk [79]
	Surplus dairy calves at 1–19 days old from 5 commercial dairy farms in Ontario, Canada	Calves (*n* = 177) were randomly assigned to three transport duration groups: 6 h (T1, *n* = 59), 12 h (T2,* n* = 60), and 16 h (T3, *n* = 58). Fecal samples were collected at four time points: before transport (0 h), immediately after transport (AT), 24 h post-transport, and 72 h post-transport	Longer transport duration (16 h) significantly reduced *Fusobacteria* abundance compared to 6 h. *Lactobacillus* decreased while *Bacteroides* and *Butyricicoccus* increased over time, with Firmicutes declining within 72 h post-transport [80]
	Simmental calves, approximately 0.5 years old	Nasopharyngeal swabs were collected from calves (*n* = 112) at three key time points: 6 h prior to loading (Group A), immediately after unloading (Group B), and after 7-day placement (Group C)	Prior to transportation, the nasopharyngeal microbiota of calves was dominated by potential bovine respiratory disease (BRD)-related pathogens, such as *Moraxella*, *Mannheimia*, and *Acinetobacter*. After 7 days of adaptive placement, there is a noticeable decrease in the abundance of these pathogens [81]
	Simmental crossbred calves (~ 8 months old) subjected to 30 h road transportation	Calves (*n* = 60) were transported 30 h from Jilin Province to Sichuan Province, China. The AMW group (*n* = 30) received AMW supplementation (30 mL/calf/d) for 3 d before and 30 d after transportation, while the Control group (*n* = 29) received no supplementation. Nasopharyngeal swabs were collected on d −3, 30, and 60	AMW-supplemented calves showed significantly lower rectal temperatures, respiratory scores, and nasal discharge scores, with higher body weight gain. compared to controls. AMW supplementation altered nasopharyngeal microbiota composition and enhanced peripheral immunity, intestinal absorption, and lipogenesis [82]
Heat stress	Male Holstein calves from birth to 68 days of age	Calves (*n* = 60) were divided into three groups: control (*n* = 20, wire hutches outdoors with 50% plywood cover), SH (*n* = 21, wire hutches in open-sided barn without fans), or SHF (*n* = 19, wire hutches in open-sided barn with ceiling fans). Nasal swabs were collected at week 5 and week 9	On week 5, the SHF group showed significantly lower mean relative abundance of *Mycoplasma* compared to the control and SH groups, on week 9, Control calves exhibited a lower mean relative abundance of *Escherichia* compared to the SHF calves, while showing a higher mean relative abundance of *Moraxella* than those in the SH and SHF groups [83]
	Holstein calves at 1–28 days of life	Calves (*n* = 16) were divided into four groups: Control (*n* = 4); low SB (LSB, *n* = 4), control milk replacer supplemented with 0.5 g of SB (*n* = 4); medium SB (MSB, *n* = 4), control milk replacer supplemented with 1.0 g of SB *n* = 4; high SB (HSB, *n* = 4), control milk replacer supplemented with 2.0 g of SB	SB supplementation decreased the fecal densities of *E. coli* and *Enterobacteriaceae* during the thermal neutral (TN) period, but no significant effects were observed during the heat stress (HS) period [85]
	Pre-weaning Holstein calves during summer	Calves (*n* = 20) were divided into two groups based on their response to heat stress: a high HS response group (H, *n* = 10) and low HS response group (L, *n* = 10)	Calves in H group had higher rectal temperatures and respiratory rates, as well as higher serum concentrations of IL-2, HSP-70, and total fatty acids (TFA) and etc., lower abundance of ruminal *Ruminococcus* and *Olsenella*, and downregulated pathways related to pyruvate metabolism and the TCA cycle [86]

### Gut barrier development

Early life microbiota is fundamental for the development and maintenance of the host's intestinal barrier function, immune system, metabolism, central nervous system, and overall health in mammals [13–16]. Evidence from germ-free mouse models indicates that microbial colonization influences epithelial proliferation and mucus layer formation. Germ-free mice exhibit reduced cell proliferation rates compared to conventionally raised animals [88], and their colonic mucus layer is thinner, though thickness is partially restored following administration of microbial-derived lipopolysaccharides and peptidoglycans [14]. These observations suggest that microbial products can stimulate barrier-related functions, though the specific taxa and metabolites responsible remain incompletely defined.

Evidence in pre-weaning calves is more limited but supports a role for microbiota in barrier integrity. Ileal mucosa-associated bacteria correlated with markers of gut barrier function under different colostrum feeding regimens [40]. Supplementation with fecal-extracted *Lactobacillus reuteri* L81 increased the expression of tight junction proteins, including ZO-1, Claudin-1, and Occludin in the small intestine [37]. However, whether these microbial interventions directly cause improved barrier function or reflect downstream consequences of other physiological changes has not been established. Additionally, the reliance on correlation-based evidence and the limited number of studies in calves preclude definitive conclusions about causal mechanisms.

### Immune system maturation

The early intestinal microbiota plays a critical role in shaping immune system development by influencing the production of immune cells and the secretion of immune molecules. These processes are critical for regulating gut immune responses, maintaining a balanced and effective immune system that can protect against infections while maintaining tolerance to beneficial microorganisms. Studies in gnotobiotic mice show that microbial absence is linked to multiple immune defects, including increased proinflammatory T-helper cells, altered regulatory T-cell responses [13], impaired T-cell differentiation [89], underdeveloped intestinal Peyer’s patches and mesenteric lymph nodes [90], and reduced immunoglobulin A (IgA) secretion [91]. Many of these defects are reversed following microbial reconstitution [89, 92, 93], suggesting that specific microbial signals contribute to immune maturation.

Evidence in pre-weaning calves is more limited. Supplementation with *Lactobacillus reuteri* L81 and *Lactobacillus johnsonii* L29 altered serum cytokine profiles [37], and intraepithelial T-cell populations correlated with the abundance of several bacterial genera, including *Lactococcus, Streptococcus*, *Succiniclasticum, Campylobacter, Gallibacterium* and *Akkermansia* [94]. However, these correlations do not establish causation. Whether these taxa directly influence immune development or simply co-occur with other factors that drive immune maturation remains unknown. Limited controlled trials and the difficulty of producing germ-free calves preclude causal inference, requiring mechanistic validation of rodent-derived principles and identification of key taxa in ruminants.

### Central nervous system development

Research indicates that the composition of the gut microbiota and its metabolites during early life plays a pivotal role in central nervous system (CNS) development, including blood–brain barrier maturation, neurotransmitter production, and neural circuit formation [16, 95]. Studies in germ-free (GF) animals show that microbial absence alters neurotrophic factor expression and behavioral phenotypes [16, 95, 96], and dysregulation of the gut-brain axis has been associated with mood disorders in humans and rodents [97, 98]. However, the specific microbial taxa, metabolites, and signaling pathways responsible for these effects remain poorly characterized, and most evidence is correlative rather than mechanistic.

Evidence linking microbiota to CNS development in neonatal calves is extremely limited. One study reported correlations between the abundance of *Lactobacillus* spp. and *E. coli* and colonic expression of serotonin receptors HTR2B and HTR4 under extended colostrum feeding [99]. While these receptors are serotonin receptors involved in regulating gut motility and intestinal barrier function. These results suggest that feeding management may influence the interaction between gut microbiota, host endocrine functions, and the gut-brain axis in neonatal calves.

### Nutrient metabolism

Gut microbiota has been associated with nutrient digestion and metabolism in pre-weaning calves, including carbohydrate fermentation, vitamin synthesis, and short-chain fatty acid (SCFA) production [5, 100, 101]. Metagenomic profiling of colonic digesta from 2-day-old calves identified carbohydrate metabolism, amino acid metabolism, and vitamin metabolism as predominant functional pathways [100], though such gene-based predictions do not confirm active metabolic contributions. Correlations have been reported between SCFA concentrations and the abundance of bacterial genera such as *Coprococcus*, *Blautia*, and *Lachnospiraceae* [5], as well as between *Bacteroides* and *Prevotella* abundance and pathways related to carbohydrate and energy metabolism [102]. Early-life dietary interventions altered populations of fiber-degrading genera including *Blautia* and *Ruminococcus* [28], and administration of the triterpenoid glycoside Anemoside B4 correlated with increased *Prevotella* abundance and predicted metabolic functions [101]. However, the majority of studies investigating the impact of intestinal microorganisms on nutrient metabolism predominantly rely on correlation analyses and gene function annotations. Direct experimental evidence linking specific microorganisms to nutrient metabolism in pre-weaning calves remains limited.

### Intestinal health

Microbial dysbiosis in pre-weaning calves has been associated with diarrhea and gastrointestinal disorders [28, 103], which is often linked to overgrowth of *Escherichia coli* or *Salmonella* [29, 104, 105]. Interventions including nutritional supplements and probiotics have shown variable efficacy in reducing diarrheal incidence. Supplementation with sodium humate and glutamine reduced fecal scores and diarrhea incidence during the first 57 d [105], prebiotics or probiotics have been used to prevent and treat diarrhea [104, 106–109], and additional immunoglobulin G during the first two weeks decreased diarrheal risk compared to milk or milk replacer alone [109]. Probiotic supplementation has been associated with reduced incidence and duration of neonatal diarrhea [110], though efficacy varies by strain and environmental context. Studies by Wu et al. [60] and Schwaiger et al. [64] demonstrated that multispecies probiotic mixtures improved gut health and reduced diarrheal rates, yet the causal mechanisms driving these phenotypic outcomes remain incompletely defined. Proposed mechanisms include lactic acid production, niche competition, direct antagonism via antimicrobial metabolites, and immunomodulation through enhanced secretory IgA production or regulatory cytokine signaling. However, these mechanisms likely act synergistically and context-dependently, yet most studies report phenotypic outcomes without elucidating the underlying microbial functions or host pathways, underscoring the need for multi-omics approaches to link specific probiotic activities to disease resistance.

### Long-term productivity

Early-life microbial composition has been associated with long-term production outcomes in dairy and beef cattle, though causal relationships remain poorly established. In dairy cattle, lower abundance of *Fusobacterium, Ruminococcaceae* UCG-001, and *Actinobacteria* in early life were significantly correlated with reduced milk yield, milk fat, and milk protein during the first lactation [111]. Early probiotic supplementation in milk or milk replacer has been linked to improved preweaning growth performance and potential feed efficiency gains [67, 112]. Studies identifying microbial taxa associated with feed efficiency in adult beef cattle suggest that early-life microbial manipulation might influence feed conversion rates [113–115]. However, these associations are largely correlative, and whether early microbial shifts directly cause long-term productivity changes remains unclear. The temporal distance between early-life microbiota and adult performance introduces numerous confounding factors, including diet, health events, and environmental conditions. Controlled trials demonstrating that neonatal microbial interventions produce measurable improvements in adult productivity are lacking.

## Conclusion and future perspectives

### Limitations and future perspectives

Current evidence linking early-life microbiota to calf health and productivity faces significant methodological constraints. Most studies rely on 16S rRNA gene sequencing and correlative analyses, which cannot establish causation or distinguish between active microbial contributors and passive bystanders. Sample sizes are often small, longitudinal studies tracking microbiota from birth through productive life are rare, and significant heterogeneity exists across studies in management practices, dietary regimens, and analytical methods, complicating generalization. Mechanistic validation methods common in rodent research remain impractical in cattle, and cross-species extrapolation from rodents to ruminants is complicated by fundamental physiological differences.

Advancing the field requires mechanistic studies employing multi-omics approaches combined with controlled interventions to identify causal microbial taxa and metabolites. Large-scale, longitudinal cohort studies incorporating standardized protocols are needed to track microbial dynamics from birth through first lactation or market weight and enable meta-analyses. Strain-specific validation of probiotic efficacy under varied environmental and management conditions is necessary to move beyond generalized recommendations.

### Conclusion

Early-life microbial colonization influences calf health and development, and management practices including nutrition, hygiene, and microbial supplementation can modulate microbiota composition. However, translating these associations into reliable strategies for improving long-term productivity requires more rigorous experimental designs, mechanistic validation, and demonstration of economic viability. While microbiome-based management shows potential, its practical implementation in livestock production requires further development.

## Data Availability

Not applicable.

## Abbreviations

ADLIB: Ad libitum milk replacer
BRD: Bovine respiratory disease
CNS: Central nervous system
FMT: Fecal microbiota transplantation
GOS: Galactooligosaccharide
HS: Heat stress
IgA: Immunoglobulin A
LFMT: Low-concentration FMT group
MOS: Mannan-oligosaccharides
PCR-SSCP: PCR single strand conformation polymorphism
SCB: Saccharomyces cerevisiae boulardii
SCFA: Short-chain fatty acid
VFA: Volatile fatty acid
